# Size Effect of Graphene Oxide on Graphene-Aerogel-Supported Au Catalysts for Electrochemical CO_2_ Reduction

**DOI:** 10.3390/ma16217042

**Published:** 2023-11-05

**Authors:** Shuling Shen, Xuecong Pan, Jin Wang, Tongyu Bao, Xinjuan Liu, Zhihong Tang, Huixin Xiu, Jing Li

**Affiliations:** School of Materials and Chemistry, University of Shanghai for Science and Technology, Shanghai 200093, China

**Keywords:** size effect, graphene oxide, graphene aerogel, electrochemical CO_2_ reduction

## Abstract

The lateral size of graphene nanosheets plays a critical role in the properties and microstructure of 3D graphene as well as their application as supports of electrocatalysts for CO_2_ reduction reactions (CRRs). Here, graphene oxide (GO) nanosheets with different lateral sizes (1.5, 5, and 14 µm) were utilized as building blocks for 3D graphene aerogel (GA) to research the size effects of GO on the CRR performances of 3D Au/GA catalysts. It was found that GO-L (14 µm) led to the formation of GA with large pores and a low surface area and that GO-S (1.5 µm) induced the formation of GA with a thicker wall and isolated pores, which were not conducive to the mass transfer of CO_2_ or its interaction with catalysts. Au/GA constructed with a suitable-sized GO (5 µm) exhibited a hierarchical porous network and the highest surface area and conductivity. As a result, Au/GA-M exhibited the highest Faradaic efficiency (FE) of CO (FE_CO_ = 81%) and CO/H_2_ ratio at −0.82 V (vs. a Reversible Hydrogen Electrode (RHE)). This study indicates that for 3D GA-supported catalysts, there is a balance between the improvement of conductivity, the adsorption capacity of CO_2_, and the inhibition of the hydrogen evolution reaction (HER) during the CRR, which is related to the lateral size of GO.

## 1. Introduction

Graphene has garnered significant attention over the past decades due to its large specific surface area, outstanding electrical and thermal conductivity, superior mechanical strength, and excellent chemical stability [1,2,3,4,5]. Three-dimensional (3D) graphene is a new type of carbon nanomaterial constructed from graphene nanosheets at the macro scale. It can maintain the excellent characteristics of graphene, overcome the π–π force between graphene layers, effectively block the self-disordered stacking of graphene nanosheets, and achieve the stability of its macro structure [6,7,8,9,10]. The structural characteristics of 3D graphene materials have shown unique advantages in applications such as electrochemical catalysis [11,12,13,14], energy storage [15,16,17,18], sensors [19,20], biomaterials [21,22,23], and environmental fields [24,25,26] and are also considered a new type of functional material with great potential. It has been found that the characteristics and prospective utilization of 3D graphene are critically influenced by the lateral size of graphene nanosheets. Generally, it is believed that large graphene nanosheets possess higher conductivity and thermal conductivity and strong mechanical properties. Small graphene nanosheets result in higher specific surface areas and active sites. For example, Kim and co-workers reported that Pt supported on smaller reduced GO (RGO) exhibited superior catalytic activities compared to those on larger RGO and the commercial Pt/C catalyst [27]. Shen and his team found that GO aerogels comprising larger sheets, approximately 30 µm in size, displayed the highest adsorption rate for cationic dyes [28].

The electrocatalytic CO_2_ reduction reaction can convert CO_2_ into a series of economically valuable reduction products under relatively mild conditions and has received widespread attention from the scientific and industrial communities in recent years [29,30,31,32,33,34]. Although great developments have been made in the CRR, there are still some challenges: the unavoidable competitive reaction, the hydrogen evolution reaction (HER), which forms high-order polycarbonate products with high overpotential and low product selectivity, and CO_2_ mass transfer limitations. Three-dimensional graphene with a porous network has been used in an electrocatalytic CRR system as a support to improve the dispersion of catalysts and the CO_2_ adsorption capacity, provide more catalytic active sites, and enhance the conductivity and stability of the catalytic system [35,36,37,38]. It has also been reported that the hydrophobicity and porous structures of supports can effectively inhibit the competitive HER [39]. Although some researchers have focused on the effect of the graphene size on 3D graphene’s performance, the influence of the graphene size on CRR performance is rarely mentioned. In this study, three GA-based nanocatalysts (Au/GA) were synthesized to research the size effect of graphene nanosheets on the CRR performance of Au/GA nanocatalysts. First, three differently sized GOs were prepared as precursors of GA. Au nanoparticles (NPs), as electrocatalysts, were decorated on the walls of the GA to synthesize Au/GA nanocatalysts via a hydrothermal method. To examine the differences of GO nanosheets in structure and morphology, AFM, XRD, FTIR, and Raman spectroscopy were performed. The properties of three Au/GA nanocatalysts were also tested using SEM, XRD, Raman spectroscopy, and EIS. The size effect of GO nanosheets on the CRR performances of Au/GA nanocatalysts was evaluated, and the mechanism was proposed.

## 2. Materials and Methods

### 2.1. Chemicals

The flake graphite (FG) powder (35 µm) used for the synthesis of large graphene oxide (GO-L) was bought from Aladdin Reagent Database Inc., Shanghai, China. The microcrystalline graphite (MG) powder (5 µm) used for the preparation of small graphene oxide (GO-S) was purchased from Chenzhou Botai Graphite Co., Ltd., Hunan, China. Sulfuric acid (H_2_SO_4_), sodium nitrate (NaNO_3_), potassium permanganate (KMnO_4_), hydrogen peroxide (H_2_O_2_), hydrochloric acid (HCl), and chloroauric acid (HAuCl_4_·4H_2_O) were all purchased from Sinopharm Chemical Reagent Co., Ltd., Shanghai, China. All the chemicals were analytical-grade and were used as received without further purification.

### 2.2. Preparation of GO-L

GO-L was synthesized via a modified Hummer’s method [40]. Typically, 2.5 g of a high-temperature expanded FG powder was weighed and added to a 1000 mL beaker, and 200 mL of concentrated H_2_SO_4_ was added. The mixture was kept at 0 °C (in an ice bath) and stirred for 25 min. Then, 10 g of KMnO_4_ was slowly introduced to the mixture over a 30 min period with continued stirring at 0 °C and was kept for 10 min. Further, the beaker was kept at room temperature for 15 min and stirred. Then, the temperature was raised to 60 °C and maintained for 4 h. After the reaction was completed, the beaker was cooled naturally to room temperature. Then, 50 mL of deionized water was dropped into the mixture and kept for 5 min at 0 °C before the addition of 150 mL of deionized water. The solution turned brown-black during this process. Next, 50 mL of 30 wt.% H_2_O_2_ was introduced to the beaker, during which the solution gradually changed from brown-black to yellow. Finally, 100 mL of a 10 wt.% dilute HCl solution was slowly added to remove excess H_2_O_2_. The obtained GO solution was subjected to multiple washes with deionized water until it reached a pH of 7.

### 2.3. Preparation of Medium-Sized Graphene Oxide (GO-M)

The medium-sized GO was purchased from Angstron Materials Inc. The purchased GO was further purified as follows: The GO solution was first centrifuged at 9000 rpm for 30 min. The supernatant underwent an additional centrifugation step at 11,000 rpm for 30 min, and the precipitate was saved.

### 2.4. Preparation of GO-S

The preparation of GO-S was according to our previous report [41]. In a typical process, 5 g of MG powder and 2.5 g of NaNO_3_ were weighed separately, added to a beaker, and mixed with 115 mL of concentrated H_2_SO_4_. The mixture was dispersed via ultrasound for 25 min and further stirred for another 25 min under low-temperature conditions (below 10 °C). Then, 15 g of KMnO_4_ was added to the mixture over 45 min with continuous stirring at 0 °C. After adding KMnO_4_, the mixture was further stirred for 25 min, accompanied by the gradual change of the solution’s color from black to dark green. Then, the temperature of the reaction system was raised to 35 °C and maintained for 45 min, accompanied by a gradual change in the solution’s color from dark green to yellowish brown during this process. The temperature of the mixture was further raised to 98 °C and maintained for 45 min. After that, the mixture was allowed to naturally cool to room temperature, and excess H_2_O_2_ was added to remove excess KMnO_4_. The excess H_2_O_2_ was removed by adding HCl. Finally, the product was rinsed with deionized water until reaching a pH of 7. The prepared GO solution was centrifuged at 16,000 rpm, and the precipitate was kept. 

### 2.5. Preparation of Au/GA

With constant stirring, 4 mL of a chloroauric acid solution (10 mg/mL) was slowly introduced to the GO solution. Next, 1.75 g of glucose was added, and the solution was stirred for another 10 min. The mixture was subsequently moved to a 50 mL Teflon-lined autoclave and maintained at 120 °C for 8 h to obtain the 3D GA loaded with Au NPs. The synthesized sample was rinsed with deionized water 6 times and soaked overnight. Finally, the samples were dried with a freeze dryer. The final samples with the GO-L, GO-M, and GO-S precursors were named Au/GA-L, Au/GA-M, and Au/GA-S, respectively. In order to eliminate the influence of the Au content on the performances of the Au/GA samples, in the above synthesis process, the Au contents in the series of Au/GA samples were controlled at 36 ± 0.2%.

### 2.6. Characterization

Atomic force microscope (AFM) images were used to test the lateral size and thickness of GO precursors using an atomic force microscope (NanoManVS, Bruker, Luken, Germany). Scanning electron microscope (SEM) images of the samples were observed using a Hitachi S4800 field emission scanning electron microscope. The microstructures of the samples were investigated using an adsorption apparatus (ASAP-2020, Micromeritics, Atlanta, GA, USA). The crystal phase of the samples was measured on a Bruker D8 Advance powder X-ray diffractometer (XRD) with Cu Kα radiation at 0.15418 nm. The Fourier transform infrared spectra (FTIR) were tested in the range of 500–4000 cm^−1^ with a Spectrum 100 spectrometer (PerkinElmer, Waltham, MA, USA). The Raman analysis of the samples was conducted using a Raman spectrometer (Horiba, LabRAM HR Evolution, Vénissieux, France) with a laser wavelength of 532 nm. CO_2_ temperature programmed desorption (CO_2_-TPD) measurements were performed on a Micromeritics AutoChem II 2920 apparatus. The contents of Au in a series of Au/GA samples were determined via inductively coupled plasma mass spectrometry (ICP-MS) (Nexion, PerkinElmer, Waltham, MA, USA).

### 2.7. Electrochemical Measurements

Preparation of the electrode: First, 2 mg of a catalyst was mixed with 50 μL of ethanol and 50 μL of a Nafion solution. The mixture was ground into a uniform paste in a mortar. Then, the paste was evenly coated onto carbon paper (1 cm^2^). Finally, the coated carbon paper was dried in an oven at 60 °C for 12 h to obtain the working electrode. The catalyst loading on the working electrode was 2 mg/cm^2^. 

All electrochemical tests, including LSV and EIS, were conducted using an Interface1000 (Gamry, Philadelphia, PA, USA) electrochemical workstation. A Pt electrode and an Ag/AgCl electrode were adopted as a counter electrode and a reference electrode, respectively. The test was conducted in a 0.1 M KHCO_3_ electrolyte in a saturated N_2_ atmosphere (repeatedly purged with high-purity nitrogen for 30 min). The scanning speed of the LSV curve was 25 mV/s. The scanning range was −0.3 V~−1.8 V vs. Ag/AgCl. The flow rate of CO_2_ during the constant potential electrolysis experiment was 30 sccm.

## 3. Results and Discussion

### 3.1. GO Precursors

**Figure 1:**
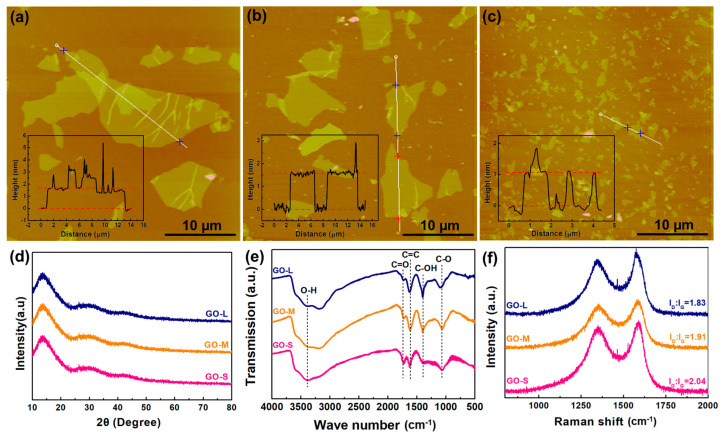
AFM images and sizes of (**a**) GO-L, (**b**) GO-M, and (**c**) GO-S. (**d**) XRD patterns and (**e**) FTIR and (**f**) Raman spectra of GO-L, GO-M, and GO-S.

GO precursors with varying sizes were selected as the building blocks to fabricate GA supports. The lateral sizes and thicknesses of these GO precursors were initially determined via AFM. As depicted in Figure 1a–c, the lateral sizes of the GO precursors exhibited clear differences. Specifically, GO-L possessed the largest lateral size, measuring approximately 14 ± 5 μm. GO-M and GO-S had average lateral sizes of 5 ± 3 μm and 1.5 ± 0.7 μm, respectively. The XRD patterns of the GO precursors were comparable, all displaying a distinct peak at around 13° (Figure 1d). This characteristic peak was attributed to the diffraction of the (001) plane of GO, resulting from the influence of oxygen functional groups, which led to an interlayer spacing of 0.68 nm. The presence of oxygen functional groups was further confirmed by the FTIR spectra (Figure 1e). As the lateral size of the GO nanosheets decreased, the characteristic peaks of the oxygen functional groups became more pronounced. This was attributed to the higher degree of oxidation in smaller GO, which resulted in more exposed edges. Consequently, the I_D_/I_G_ ratio (area intensity ratio of the D band to the G band; Figure S1), which served as a measure of the disorder degree of GO, increased with a decreasing lateral size of GO (Figure 1f and Table S1). The significant differences in size between the GO precursors were sufficient to study the size effect of GO on the performances of GA supports.

### 3.2. Au/GA Nanocatalysts

**Figure 2:**
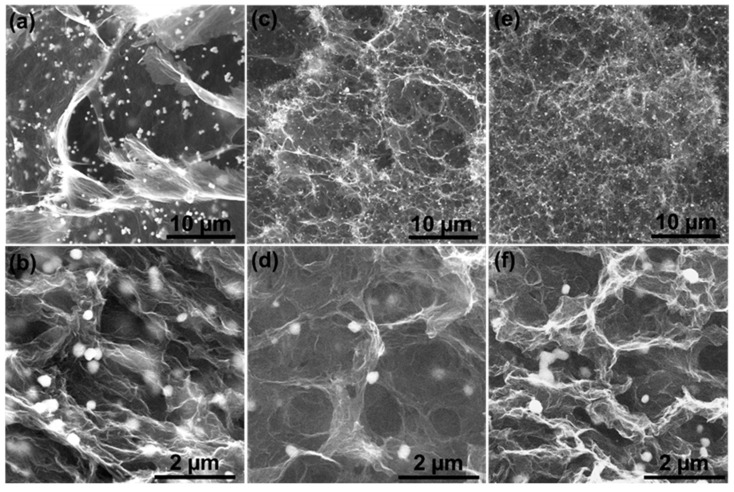
SEM images of (**a**,**b**) Au/GA-L, (**c**,**d**) Au/GA-M, and (**e**,**f**) Au/GA-S.

After the hydrothermal treatment of the GO and Au precursor, Au/GA nanocomposites were obtained. SEM was adopted to observe the inner morphologies of the resulting Au/GA. As shown in Figure 2a,b, GO-L stacked more closely together, leaving fewer gaps and big pores in Au/GA-L. Au NPs were wrapped in the connected GO sheets. Au/GA-M exhibited an obviously porous network. Au NPs uniformly decorated the walls of connected pores (Figure 2c,d). The Au/GA-S prepared with the smallest GO-S (~1.5 µm) displayed thicker walls and smaller pores (Figure 2e,f). Some of the small pores were isolated. The hydrothermal method for GA synthesis from GO involves the partial reduction of GO sheets, their mutual adhesion, gradual contraction to form graphene hydrogels, and subsequent processing to obtain GA [42,43]. The size of the initial GO precursors plays a significant role in determining the resulting microstructure of GA. Larger sheets lead to fewer pores, and smaller sheets favor a more porous network. However, insufficient GO nanosheets cause excessive stacking between layers, reduce the specific surface area, and increase the wall thickness.

**Figure 3:**
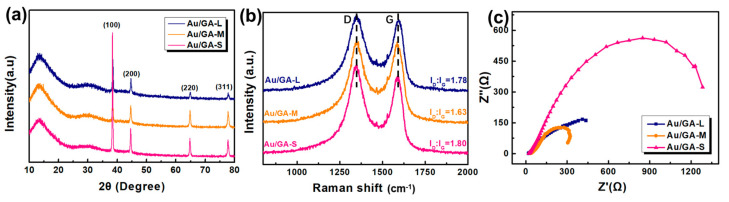
(**a**) XRD patterns, (**b**) Raman spectra, and (**c**) Nyquist plots of Au/GA-L, Au/GA-M, and Au/GA-S.

The XRD patterns of the Au/GA samples displayed distinct diffraction peaks at 38°, 44°, 65°, and 78°, corresponding to the (111), (200), (220), and (311) crystallographic planes of Au, respectively (Figure 3a). This result indicates that all Au/GA samples with different pore structures contained Au, which is consistent with the SEM results. Raman characterizations of three Au/GA samples were performed. The ratios of I_D_:I_G_ for Au/GA-L, Au/GA-M, and Au/GA-S were 1.78, 1.63, and 1.80, respectively (Figure 3b and Figure S2). These ratios were lower than those of their corresponding GO precursors (Table 1), suggesting the reduction of oxygen groups due to the hydrothermal treatment. The lower the number of defects, the more favorable the transmission of electrons.

EIS was used to characterize the charge transfer ability of Au/GA samples as an auxiliary measurement to evaluate the catalytic properties of the electrocatalyst. A higher resistivity of the working electrode indicated higher energy consumption and worse catalytic performance; a lower resistivity of the working electrode indicated a higher charge transfer capability of the electrode and better catalytic performance. As shown in Figure 3c, Au/GA-M exhibited the smallest impedance, suggesting it had the strongest electron transfer capacity. As expected, Au/GA-S displayed the largest impedance, as it had the highest degree of defects (Table 1).

### 3.3. CRR Performance

**Figure 4:**
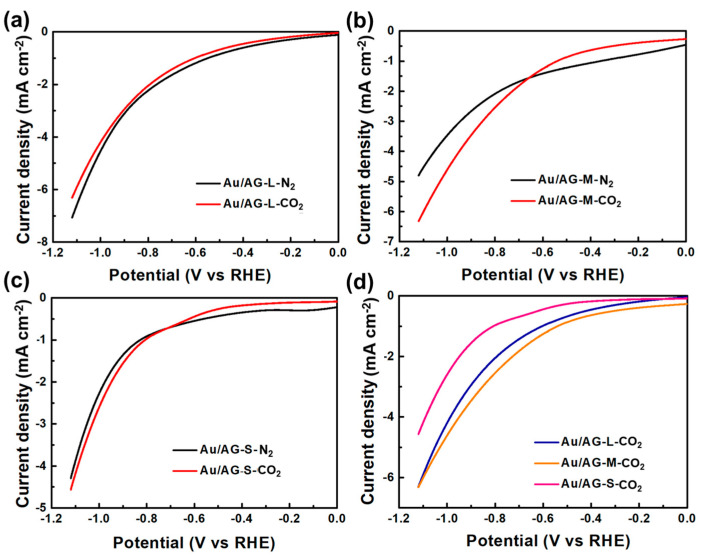
LSV curves of (**a**) Au/GA-L, (**b**) Au/GA-M, and (**c**) Au/GA-S and (**d**) current densities of three samples in 0.1 mol/L KHCO_3_ electrolytes.

LSV curves show the response degree of a catalyst to a current at different potentials to preliminarily determine the electrocatalytic activity of a catalyst. As shown in Figure 4, three Au/GA nanocatalysts with different pore structures responded to different current densities at different potentials. The LSV curves of Au/GA-L indicate that the current density in the saturated N_2_ atmosphere was slightly higher than that in the saturated CO_2_ atmosphere, suggesting that Au/GA-L may prefer the HER rather than the CRR. The current density of Au/GA-M in the saturated N_2_ atmosphere before −0.69 V was higher than that in the saturated CO_2_ atmosphere, indicating that Au/GA-M may prefer the HER before this potential. Nevertheless, the current density in the saturated CO_2_ atmosphere after −0.69 V was higher than in the saturated N_2_ atmosphere, indicating that Au/GA-M starts to transition to the CRR after this potential. Similarly, Au/GA-S also began to prefer the CRR at around −0.69 V. Figure 4d shows that the reduction current density of Au/GA-M in the saturated CO_2_ atmosphere was slightly higher than that of Au/GA-L and much higher than that of Au/GA-S. The above results indicate that the electrocatalytic activity of Au/GA-M is theoretically higher than those of Au/GA-L and Au/GA-S.

**Figure 5:**
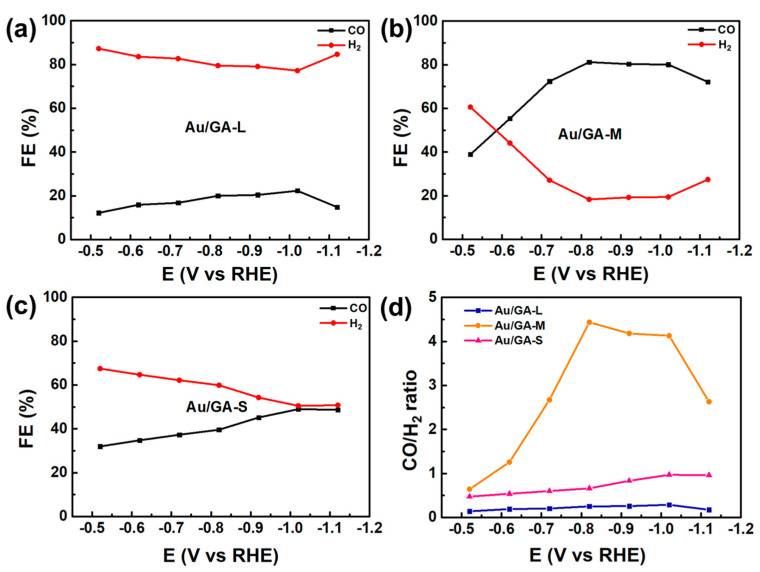
(**a**–**c**) FEs and (**d**) CO/H_2_ ratios of Au/GA-L, Au/GA-M, and Au/GA-S at different potentials.

**Figure 6:**
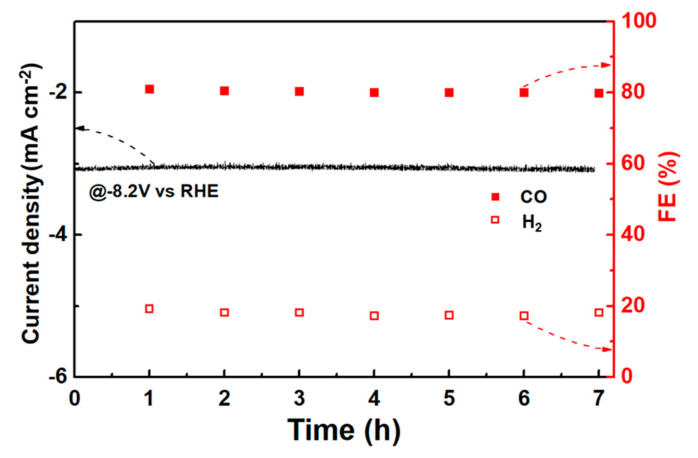
Stability analysis of Au/GA-M over 7 h at -8.2 V.

The products of the CRR of the three samples could be detected online using a gas chromatograph connected to an electrochemical workstation. Since the main CRR product of the noble metal Au is CO, the main products CO and H_2_ were pertinently detected, and Figure 5 presents the detection results. As expected, Au/GA-M showed the optimal ability to transform CO_2_ to CO. The Faradaic efficiency (FE) of CO reached a maximum value of 81% at −0.82 V vs. an RHE. This was followed by Au/GA-S, with a maximum value of 46% at −1.02 V vs. an RHE, and finally Au/GA-L, with a maximum value of only 20% at −1.02 V vs. an RHE (Figure 5a–c). Although Au/GA-L responded more to a current with the same potential and had a stronger electron conduction capacity than Au/GA-S (Figure 3c and Figure 4d), its CRR performance was much lower than those of the other samples. For Au/GA-L, it preferred the HER rather than the CRR, and thus the CRR performance of Au/GA-L was lower than that of Au/GA-S (Figure 5d). Specifically, the CO/H_2_ ratio of Au/GA-M reached a maximum of 4.4 at −0.82 V. The stability test of Au/AG-M revealed that the catalytic performance of Au/AG-M for CO showed no significant decline during a 7 h reaction, with an FE_CO_ consistently exceeding 80% and an FE_H2_ lower than 20% (Figure 6). This demonstrates that the Au/AG-M catalyst maintains both high catalytic activity and stability throughout the CRR process.

**Figure 7:**
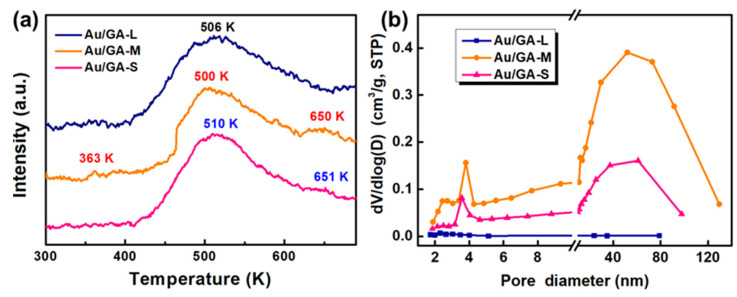
(**a**) CO_2_-TPD analysis and (**b**) pore size distribution of Au/GA-L, Au/GA-M, and Au/GA-S.

CO_2_-TPD characterizations of three Au/GA samples were carried out. In general, the desorption peak of CO_2_ can be divided into three regions: low temperature (300~400 K), medium temperature (400~600 K), and high temperature (600~800 K), corresponding to weak, moderate, and strong adsorption to CO_2_, respectively. As shown in Figure 7a, only Au/GA-M had CO_2_ desorption peaks in all three temperature regions, indicating it had the highest CO_2_ adsorption capacity. Au/GA-S had CO_2_ desorption peaks in the medium-temperature region and high-temperature region. Au/GA-L only had CO_2_ desorption peaks in the medium-temperature region, and thus the adsorption capacity of CO_2_ was inferior to that of Au/GA-M. It is worth noting that among the three samples, Au/GA-M had the highest adsorption capacity for CO_2_ and the strongest capability to convert CO_2_ into CO. Au/GA-S had an intermediate adsorption capacity for CO_2_ and a moderate capability to convert CO_2_ into CO. Au/GA-L had the worst adsorption capacity for CO_2_ and the worst capability to convert CO_2_ into CO. Therefore, it can be inferred that the three samples had different CRR properties resulting from different adsorption capacities for CO_2_, which related to the microstructures of Au/GA.

For a precise assessment of the microstructures of these Au/GA samples, the texture properties were explored via nitrogen sorption experiments. As shown in Figure 7b, Au/GA-L displayed no pores within the mesoporous range because the pores in Au/GA-L were larger (Figure 2a). Au/GA-S and Au/GA-M contained certain amounts of mesopores and macropores. Notably, the Au/GA-M sample exhibited pore sizes of 2.3 nm, 3.9 nm, and 55 nm, indicating the presence of a hierarchical pore structure. The presence of mesopores provided a larger surface area and higher pore volumes (Table 2). Smaller GO nanosheets are more prone to the formation of isolated voids, which is detrimental to the diffusion of CO_2_ molecules.

Based on the above findings, the reason for the effect of the lateral size of GO precursors on the CRR performance of Au/GA is discussed. It is well known that the adsorption and diffusion rates of CO_2_ molecules vary with the pore sizes of porous materials. For Au/AG-L, the size of the GO precursor was larger, the pore diameter of the GA was larger, and the structure of Au/AG-L was relatively loose. The larger pore size meant a higher diffusion rate. CO_2_ molecules could pass more easily through larger channels with less interactions between CO_2_ molecules and catalysts. For Au/AG-S, the size of the GO precursor was the smallest. The denser structure of Au/GA-s led to isolated pores, limiting the transport of CO_2_ molecules in the material. Au/AG-M had a hierarchical pore structure and the largest specific surface area, which facilitated the diffusion of CO_2_ and interactions with the catalysts.

## 4. Conclusions

In conclusion, three Au/GA electrocatalysts were prepared via a hydrothermal method using three different-sized GO nanosheets and chloroauric acid as precursors. Au NPs all dispersed uniformly on the walls of 3D GA for the three Au/GA catalysts. Au/GA-M, constructed with a medium-sized graphene oxide (~5 µm), exhibited a hierarchical porous network and the highest surface area and conductivity, which were favorable for the adsorption and diffusion of CO_2_ molecules in the 3D structure. As a result, Au/GA-M exhibited the highest FE_CO_ of 81%. Furthermore, the three Au/GA samples exhibited a tunable CO/H_2_ ratio, which is beneficial for the electrocatalytic conversion of CO_2_ to synthesis gas. This study demonstrates that the CRR performance of 3D graphene-based electrocatalysts can be tuned by modulating the lateral size of graphene nanosheets to balance the conductivity, the adsorption capacity of CO_2_, and the inhibition of the HER.

## Figures and Tables

**Table 1:** I_D_:I_G_ ratios of Au/GA-L, Au/GA-M, and Au/GA-S and their corresponding GO precursors.

Samples	GO-L	GO-M	GO-S	Au/GA-L	Au/GA-M	Au/GA-S
I_D_:I_G_ ratio	1.83	1.91	2.04	1.78	1.63	1.80

**Table 2:** Specific surface areas and pore volumes of Au/GA-L, Au/GA-M, and Au/GA-S.

Samples	Au/GA-L	Au/GA-M	Au/GA-S
BET surface area (m²/g)	15	98	45
Pore volume (m^3^/g)	0.01	0.37	0.17

## Data Availability

Data is contained within the article or supplementary material.

## Supplementary Materials

The following supporting information can be downloaded at: https://www.mdpi.com/article/10.3390/ma16217042/s1.
