# Supplementary material for: Size Effect of Graphene Oxide on Graphene-Aerogel-Supported Au Catalysts for Electrochemical CO2 Reduction
Source: Materials (Basel). 2023 Nov 5;16(21):7042. doi: 10.3390/ma16217042 (PMC10650518; doi:10.3390/ma16217042)
Supplement: Supplementary file 1 [file materials-16-07042-s001.zip › materials-2689475-supplementary.pdf]

Supporting Information

Size Effect of Graphene Oxide on Graphene-Aerogel-Supported Au Catalysts for Electrochemical CO_2 Reduction

Shuling Shen,* Xuecong Pan, Jin Wang, Tongyu Bao, Xinjuan Liu, Zhihong Tang, Huixin Xiu, Jing Li*

School of Materials and Chemistry, University of Shanghai for Science and Technology, Shanghai, 200093, China

* Correspondence: slshen@usst.edu.cn (S.S.); lijing6080@usst.edu.cn (J.L.)

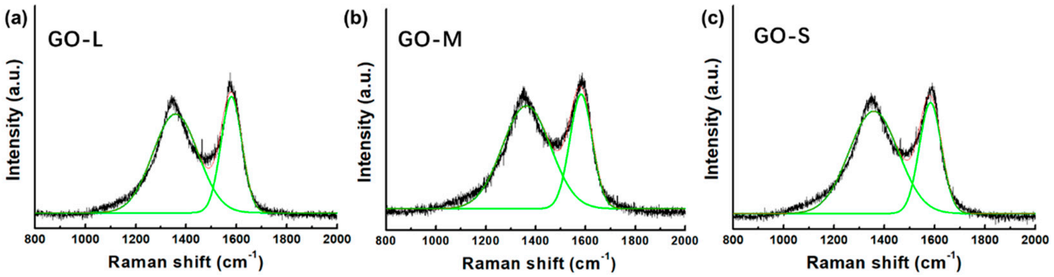

Figure S1. Raman spectra fitting results of (a) GO-L, (b) GO-M, (c) GO-S.

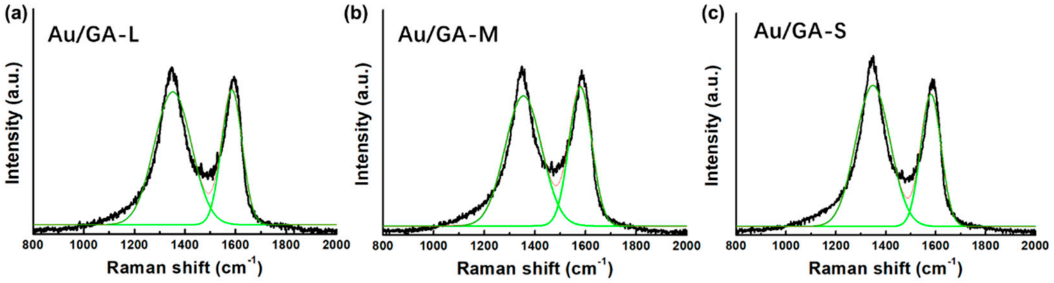

Figure S2. Raman spectra fitting results of (a) Au/GA-L, (b) Au/GA -M, (c) Au/GA -S.

Table S1. The areas values of D band and G band of the obtained samples measured from Raman results.

Samples	I_D	I_G	I_D:I_G
GO-L	31840.8	17441.7	1.83
GO-M	27059.9	14131.7	1.91
GO-S	41564.6	20370.7	2.04
Au/GA-L	132723.2	74634.1	1.78
Au/GA-M	137852.5	84572.1	1.63
Au/GA-S	145057.5	80772.5	1.80
